# Measurement of junctional tension in epithelial cells at the onset of primitive streak formation in the chick embryo via non-destructive optical manipulation

**DOI:** 10.1242/dev.175109

**Published:** 2020-02-04

**Authors:** Valentina Ferro, Manli Chuai, David McGloin, Cornelis J. Weijer

**Affiliations:** 1 Department of Physics, School of Science and Engineering, University of Dundee, Dundee DD1 4HN, UK; 2 School of Life Sciences, University of Dundee, Dundee DD1 5EH, UK; 3 School of Electrical and Data Engineering, University of Technology Sydney, Ultimo, NSW 2007, Australia

**Keywords:** Gastrulation, Optical tweezers, Primitive streak, Tension

## Abstract

Directional cell intercalations of epithelial cells during gastrulation has, in several organisms, been shown to be associated with a planar cell polarity in the organisation of the actin-myosin cytoskeleton and is postulated to reflect directional tension that drives oriented cell intercalations. We have characterised and applied a recently introduced non-destructive optical manipulation technique to measure the tension in individual epithelial cell junctions of cells in various locations and orientations in the epiblast of chick embryos in the early stages of primitive streak formation. Junctional tension of mesendoderm precursors in the epiblast is higher in junctions oriented in the direction of intercalation than in junctions oriented perpendicular to the direction of intercalation and higher than in junctions of other cells in the epiblast. The kinetic data fit best with a simple viscoelastic Maxwell model, and we find that junctional tension, and to a lesser extent viscoelastic relaxation time, are dependent on myosin activity.

## Abstract (precis)

**Summary:** Optical tweezing of cell junctions in the epiblast of early gastrulation chick embryos shows that junctional directional tension is coincident with myosin planar cell polarity and directional cell intercalation.

## INTRODUCTION

**Fig. 1.**
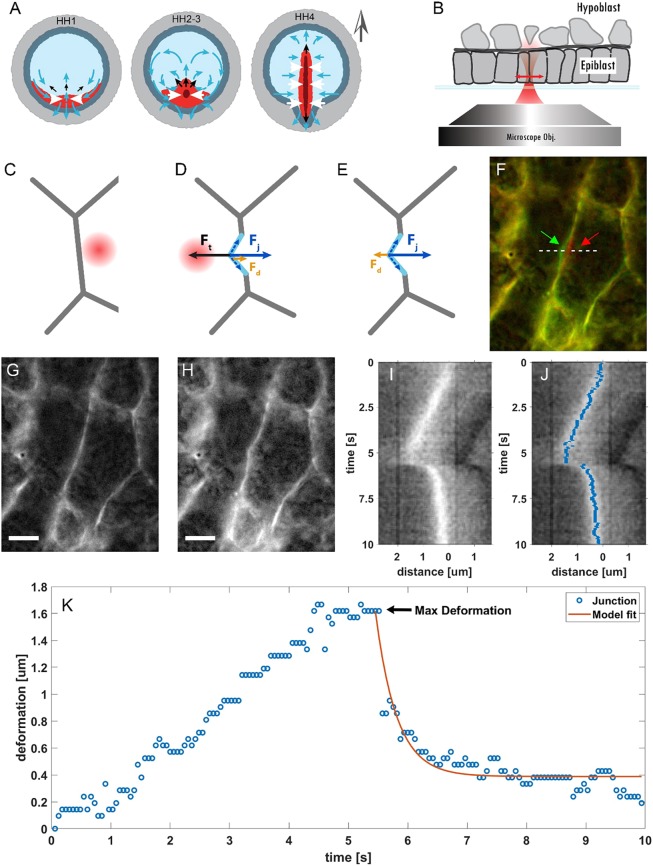
**Optical manipulation of cell-cell junctions.** (A) Stages 1-4 of chick embryo development according to Hamburger and Hamilton (1992). The different regions in the embryo are shown with different colours. The central part of the embryo, known as the area pellucida (light-blue region) will form the embryo proper and is separated from the extra-embryonic region, the area opaca (light grey region), by the marginal zone (dark grey region). The presumptive mesendoderm (red region) is located in the posterior area of the embryo next to the marginal zone and will form the streak. At stage EGK XIV, the mesendoderm cells start to move (blue arrows) due to active pulling forces (white arrows) generated in this tissue. The contraction of this tissue generates pushing forces (black arrows) that result in elongation of the streak at stages HH2-3. From stage HH3 onwards, the mesendoderm cells start to ingress into the embryo through the streak. The grey arrow outside the embryo indicates the A-P axis. (B) Schematic of the experiment in a cross-sectional view: the chick embryo is situated on a glass-bottom plate with the epiblast facing the microscope objective. The optical trap is moved perpendicular to the cell-cell junctions (double-headed red arrow). (C-E) Bottom view of the experiment: the trap is turned on while on the right side of a selected junction (C) and then moved across the junction; once the trap crosses the junction, it deflects it (D). The maximum deformation is obtained when the optical force F_t_ is balanced by the tension of the junction F_j_ and the drag in the cytosol F_d_. When the trap is turned off (E), F_j_ restores the junction to its rest position. The force diagrams reflect the geometry of local junctional deformation observed in some of the experiments (F,H). In other cases, the deformations extend across the full length of the junction. (F-H) False-colour image corresponding to two time frames (F). The red and green arrows point to the deformation of the junction before and after pulling. The red channel is the junction at rest position at t=0 (G); the green channel is the junction at its maximum deformation (H). The images are extracted from Movie 1. Scale bars: 5 µm. (I) Kymograph of the junction deformation collected at the row indicated by the white dashed line in F. (J) Superposition of kymograph in I and the junction position extracted by the seam-carving algorithm (blue pixels). (K) Junction position as extracted from the kymograph and corresponding fit using the viscoelastic model (Eqn 5).

Development is characterised by the formation and shaping of new tissues of increasing complexity. An early crucial phase of tissue formation is gastrulation where the main three germ layers, the ectoderm, mesoderm and endoderm, are formed and take up their correct topological positions in the embryo (Stern, 2004). In amniote embryos, gastrulation starts with the formation of the primitive streak, the structure through which the mesoderm and endoderm precursors move into the embryo (Antin et al., 2007; Bachvarova et al., 1998). The process of streak formation has been widely studied in chick embryos, as these can be cultivated *in vitro* and are readily accessible to manipulation (Stern, 2004). The streak starts to form in the posterior part of the epiblast and extends in the anterior direction during its formation. Streak formation has been shown to involve large scale vortex-like tissue flows in the epiblast (Chuai et al., 2006; Cui et al., 2005; Gräper, 1929; Vakaet, 1970; Voiculescu et al., 2007). The vortex flows initiate in a sickle-shaped area of the posterior epiblast that gives rise to the endoderm and mesoderm (Fig. 1A). Using a recently developed transgenic chick strain, in which the cell membranes are labelled with GFP, and a dedicated lightsheet microscope, we have previously been able to observe the process of streak formation at both the tissue and cellular level in great detail (Rozbicki et al., 2015).The cellular mechanisms that have been proposed to drive these flows involve directed cell shape changes and cell intercalations, and are supported by cell divisions and ingression of individual cells in the epiblast (Firmino et al., 2016; Rozbicki et al., 2015; Voiculescu et al., 2014). Before the onset of the tissue flows, the mesendoderm precursor cells are elongated and aligned in the direction of the forming streak. The onset of motion is correlated with cell shape changes and cell intercalations perpendicular to the anterior-posterior (A-P) axis in the mesendoderm. Aligned cells form transient chains of junctions and these junctions are enriched in active myosin, as detected by phosphorylation of the myosin light chain (Rozbicki et al., 2015). Blocking of myosin II activity relaxes cell shapes and inhibits directional cell intercalations and streak formation. Further experiments have shown that blocking of myosin I results in a relaxation of the cells and absence of the formation of myosin II cables in aligned cell junctions (Rozbicki et al., 2015).

The cellular behaviours observed during gastrulation in the chick embryo thus appear to show very strong similarities to those that drive gastrulation in *Drosophila*, a process that involves extension of the body axis and is known as germband extension (Zallen and Wieschaus, 2004). Germband extension has been shown to depend on directional cell intercalations, driven by myosin II-mediated directional shorting of junctions (Bertet et al., 2004; Blankenship et al., 2006; Guirao and Bellaïche, 2017). It has furthermore been shown by localised laser ablation experiments that the contracting junctions are under greater tension than non-contracting junctions (Fernandez-Gonzalez et al., 2009). Recently, a more quantitative non-destructive method has been developed to measure the tension in individual junctions directly by pulling on them, using a laser tweezers approach (Bambardekar et al., 2015). From the extension of the junction and the kinetics of movement of the junction as it returns to the resting state after release from the trap, it was possible to extract parameters such as stiffness and viscosity of the junction. It was shown that the stiffness is around 2.5-fold higher in contracting junctions than in parallel non-contracting junctions during germband extension, while the measured viscosity can account for long-term dissipation of tension (Clément et al., 2017). The latter was shown to be largely dependent on remodelling of the actin cytoskeleton.

Our working hypothesis to explain gastrulation in the chick embryos is that myosin II activity drives directional intercalation through the generation of directional tension. Testing this hypothesis requires measurement of junctional tension under a variety of experimental conditions. Here, we demonstrate a modified version of the optical trapping technique used to measure tension in *Drosophila* junctions (Bambardekar et al., 2015; Clément et al., 2017) and find that we can optically manipulate junctions in cells of the epiblast of early gastrulation stage chick embryos. However, we observed that, in most cases, we did not trap the junctions directly, but generally vesicles in epiblast cells, which are then used as probes that deform the membrane. We performed relative tension measurements in embryos in the early stages of streak formation and measured differences in junctional tension that varied with position in the epiblast and orientation of the junctions. Through the use of specific inhibitors, we have defined the contribution of myosin I and myosin II towards generating tension in these junctions.

## RESULTS

Mechanics, especially the tension of cell junctions, are a major determinant of cell shape, which in turn underlies tissue shape (Guillot and Lecuit, 2013). Changes in junctional tension drive a diverse array of cell behaviours. The mechanical properties of cell-cell junctions can be determined by applying external forces to the junctions while measuring their responses to these perturbations. We opted for optical tweezers to perform tension measurement in chick embryos, as this allowed us to apply optical forces to probe the system in a non-destructive manner, contrary to commonly used methods such as laser ablation, and without the need to introduce external probes, such as oil droplets in the embryos (Campàs et al., 2014; Rauzi et al., 2008). We can measure the dynamics of a junction after it is moved a given distance from its equilibrium position. The deformation, which results in increased tension in the junction, generates a restoring force. When the junction is released, the tension enables the junction to contract back to its rest position (Fig. 1) (Bambardekar et al., 2015). To monitor the deflection of the junction, we used transgenic embryos expressing a membrane-localized GFP in combination with high-resolution fluorescence microscopy.

We designed and built an instrument integrating optical tweezers in an inverted fluorescence microscopy setup (Lee et al., 2007). We used a single microscope objective to both focus the light from a high-intensity infrared laser to produce the tweezers and to illuminate the sample with excitation light suitable to excite the GFP in the cell membranes (Fig. S1).

In our experiments, the trapping laser sweeps at right angles over the junction to be trapped in a single pass, starting in a cell on one side of the junction and ending up across the junction in a neighbouring cell, after which the trap is turned off (Fig. 1B-E). We used 750 mW of laser power (measured in the image plane) and a measurement cycle of 10 s. This provided a good compromise between a small but measurable deflection of the majority of the tested junctions and the absence of any obvious visible signs of damage (Fig. S2). We observed that, when the trap is activated, the optical force overcomes the other forces acting on the junction (i.e. the tension and the drag from the cytosol) and it starts to deform (Fig. 1D,F-H). The junction reaches a maximum deformation when all the forces acting on it are in equilibrium (Fig. 1C-H). When we turn the trapping laser off, the junctional tension restores the junction to its rest state, opposed only by the drag in the cytosol (Fig. 1E). Stiffer junctions are lost from the moving trap before it reaches its final position.

To study the deformation over time, we generated kymographs of the junctions (Fig. 1I). The kymograph shows that the junction follows the tweezers until it reaches its maximum deformation. When the trap is turned off, the junction then moves back to, or close to, its original position. This return phase follows an exponential decay determined by a time constant that is characteristic of the mechanical properties of the junction and its surroundings.

To perform the analysis, we extracted the position of the junction over time by applying a seam-carving algorithm to the kymographs (Fig. 1J-K). This method is limited to pixel resolution, but we observed that, in our experiments, it outperformed other more typical approaches, such as a Gaussian fit of the image to localise the junction (Fig. S3). We then fitted the junction deformation over time and extracted the maximum deformation and the relaxation time.

### Optical trapping of cell-cell junctions

**Fig. 2.**
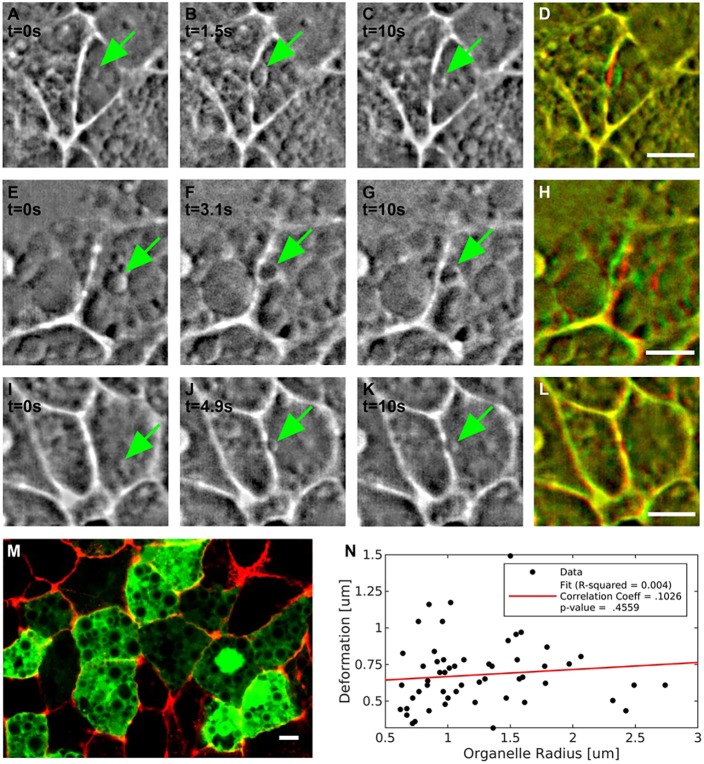
**Role of trapped organelles in the deformation of the junctions.** (A-L) Representative images of three pull-and-release experiments. In each experiment, an organelle (green arrow) is trapped and drives the deformation of the junction. (A-D) The sequence shown is extracted from Movie 2. In the three sequences (A-D, E-H and I-L), the first image in each row (A,E,I) shows the vesicle before being trapped; B,F,J show the vesicle after being pushed against the membrane; C,G,K show the vesicle at the end of the experiment when the trap is off. (D,H,L). The three sequences represent the different scenarios we observed during the experiments. In A-D, an organelle locally deforms the junction; in E-H, the deformation caused by the organelle extends across the whole junction; in I-L, the junction does not deform despite an organelle pushing against it. (M) Image of a confocal section of the area opaca epiblast of a HH1 stage embryo in which some random transfected cells express a cytosolic GFP (green) highlighting the abundance of organelles, which are visible as dark structures of different sizes in the cytosol of typical epiblast cells. The cell boundaries are visualized by actin staining (red). The cells have been transfected with a cytoplasmatic GFP; construct shows vesicles as dark spots. (N) Absence of correlation between sizes of trapped organelles and deflections of the junctions. The dataset has a correlation coefficient (r) of 0.103 and *P*=0.46, thus confirming that the measured deformations are not dependent on the sizes of trapped organelles. Scale bars: 5 µm.

The optical tweezers used to optically manipulate cell-cell membranes in living embryos (*Drosophila*) were calibrated by assuming that they would directly interact with and trap the cell-cell junction (Bambardekar et al., 2015; Clément et al., 2017). However, during our experiments in the chick embryo, we noticed that, in the majority of cases, small vesicular organelles in the proximity of junctions were trapped initially and closely followed the movement of trap. These organelles are then pushed against the junction, leading to its deformation (Fig. 2). By using a Fourier bandpass filter, we enhanced the contrast of these objects and observed how the organelles trapped by the tweezers ultimately are responsible for pushing and deforming the junctions (Fig. 2A-C,E-G,I-K). Vesicular organelles of different sizes are highly abundant in the epiblast cells of early chick embryos, as can be clearly seen in confocal images of scattered cells expressing a cytosolic GFP marker that is excluded from these organelles and thus appear dark (Fig. 2M).

The observation that, in most cases, we likely trap organelles that then push the junction plays an important role in the interpretation of the results. The stiffness of optical tweezers is dependent on the refractive index, and the shape and size of the trapped object (Neuman and Block, 2004; Montange et al., 2013). As the trapped vesicles vary in size, the stiffness of the tweezers will vary also between measurements. We tested whether a measurable significant correlation existed between the size of the vesicles and the deformation observed. We did not detect one, suggesting that other variables are likely more important than the variation in organelle size (Fig. 2N). As it is practically difficult to pull every junction exactly perpendicular to the orientation of the junction, we investigated whether there was a significant effect of the precise direction of pulling on the measured deformation. We did not detect a systematic effect in our experiments (Fig. S4A). We furthermore decided to test whether there was a significant correlation with junction length that was not detected either (Fig. S4B). Therefore, the most likely cause of the significant variation in measured deformation is an underlying variation in junctional tension. Maintaining the properties of the laser trap, such as laser power, distance travelled by the tweezers and duration of the experiment constant, we expect that higher tensions will result in smaller maximum deformation of the junctions. Based on the above reasoning and observations, we decided to use the trapping method to measure and compare the responses of junctions in different places and orientations in the embryo and under different experimental perturbations. We measured 40-100 junctions per condition and measurements were derived from three to five embryos at similar stages of development.

### High junctional tension in mesendoderm cells

**Fig. 3.**
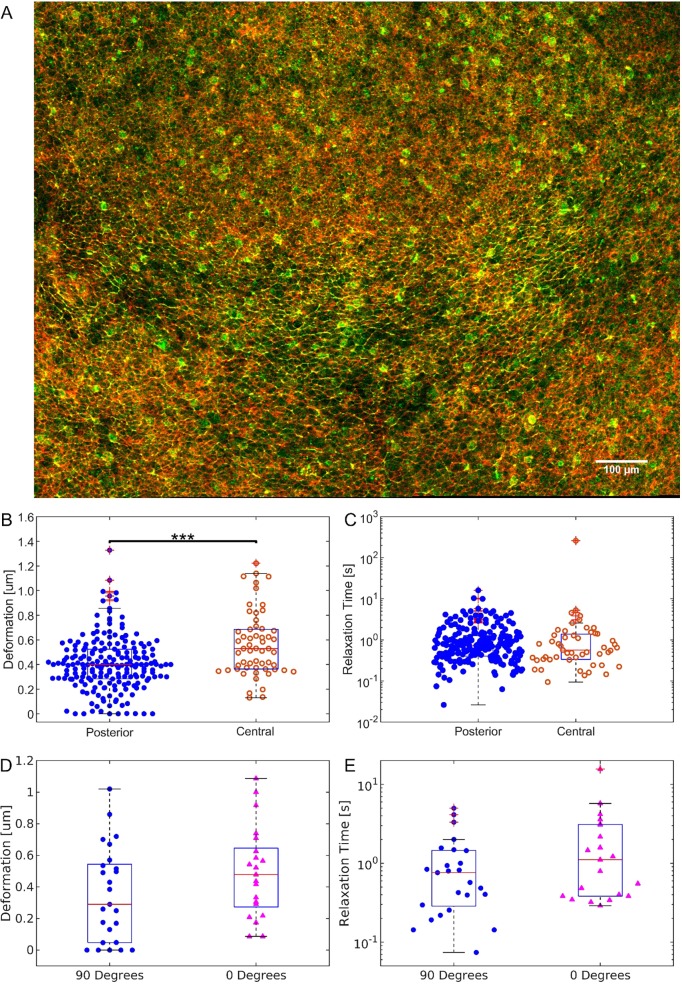
**Junction deformation and relaxation time for the posterior and the central area of the embryo.** (A) Image of the posterior part of the embryonic area of a chick embryo: posterior is towards the bottom; anterior is towards the top. The image shows phosphor-specific myosin light chain antibody staining in green and actin detected by phalloidin staining in red. The supercellular phospho-myosin cables are arranged in a horizontal arc in the mesendoderm domain located in the middle slice of the embryo. Scale bar: 100 µm. (B) Box and whisker plot and distribution of the maximum deformation of junctions measured in the posterior (blue dots) and in the central area (orange circles) of the embryo. The maximum deformation measured in the posterior area was 0.39 µm, [median, *n*=203, 90% confidence interval CI (0.13 µm, 0.65 µm)], while in junctions located outside the mesendoderm in the central area of the embryo, we measured a value of 0.58 µm (median, *n*=57, 90% CI (0.32 µm, 0.99 µm)]. The data for the posterior area were aggregated from ten different embryos, the same referred to as ‘Control’ in Fig. 4 (*n*=203). The data for the central area were aggregated from three different embryos (*n*=57). ***P<0.001. (C) Box and whisker plot and distribution of the relaxation times. The measured relaxation times for this dataset are 0.7 s [*n*=203, 90% CI (0.3 s, 3.1 s)] and 0.6 s [*n*=57, 90% CI (0.2 s, 2.8 s)] for junctions in the posterior and the central area, respectively, and are not statistically significantly different. (D) Box and whisker plot and distribution of the maximum deformation of junctions measured in the posterior (blue dots) perpendicular to the direction of streak formation (‘90 degrees’ dataset) and in the posterior of the embryo along the direction of streak formation (pink triangles, ‘0 degrees’ dataset). The average deflection is 0.35 μm (median=0.29 μm) in the direction perpendicular to the A-P axis and 0.49 μm (median=0.48 μm) in the direction parallel to the A-P axis. (*P*=0.07). (E) Deformation time constants in the direction perpendicular (blue dots) and parallel (pink triangles) to the A-P axis. The average relaxation time is 1 s (median=0.7 s) in the direction perpendicular to the A-P axis and 2 s (median=1.1 s) in the direction parallel to the A-P axis. The data for the perpendicular dataset (*n*=25) and the data for the parallel dataset (*n*=21) are pooled from measurements of two embryos (*P*=0.1). In B-E, the horizontal line represents the median, the boxes represent the 25th and 75th percentiles, the vertical bars represent the 9th and 91st percentiles, and the crosses represent outlier data points.

In chick embryos at the early stages of streak formation, preferential accumulation of active myosin occurs in junctions of mesendoderm cells that are aligned in the direction of contraction and often align with junctions of neighbouring cells to form what appear to be super-cellular myosin cables (Fig. 3A). We have hypothesized that this myosin accumulation generates a significant junctional tension that will drive the directed cell-cell intercalations that mediate the elongation of the streak (Fig. 3A) (Rozbicki et al., 2015). We first wanted to verify the hypothesis that junctions oriented perpendicular to the A-P axis in the posterior mesendoderm precursor cells in the epiblast of embryos about to form a streak (EGK XIII-XIV) are under higher tension compared with junctions elsewhere in the embryo. We therefore measured deformation in junctions aligned perpendicular to the A-P axis in the posterior mesendoderm part of the embryo and compared this with the deformation of junctions with similar orientation in the central part of the embryo before the formation of the streak. We observed a significant difference in the maximum deformation measured (Fig. 3B). Fitting the junctions with a Maxwell viscoelastic model, we extracted the relaxation time for each junction (supplementary materials, Fig. S8). The difference between the two distributions was not significant according to the Wilcoxon rank sum test (Fig. 3C). Thus, the observed differences in junction deflection amplitude of cells located in the central and the posterior areas of the embryos clearly indicate that the junctional tensions of cells in these areas are different.

**Fig. 4.**
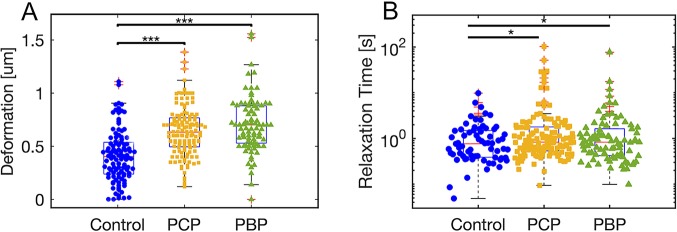
**Junction deformation and relaxation time for embryos treated with myosin inhibitors.** (A) Box and whisker plot and distribution of the maximum deformation of junctions measured in the posterior of stage EGK XIII-XIV in control embryos after treatment with 0.1% DMSO (blue dots, *n*=106) and embryos treated with 10 µM PCP (yellow squares, *n*=132) or treated with 10 µM PBP (green triangles, *n*=88), as described in the Materials and Methods. Median values for the deformations are 0.41 µm [90% CI (0.12 µm, 0.82 µm)], 0.62 µm [90% CI (0.35 µm, 0.95 µm)] and 0.71 µm [90% CI (0.35 µm, 0.95 µm)] for the control, the PCP and the PBP datasets, respectively. (B) Box and whisker plot and distribution of relaxation times. Median values for the relaxation times are 0.76 s [*n*=63, 90% CI (0.3 s, 2.6)] for the control sample and 1 s for the PCP- [*n*=132, 90% CI (0.4 s, 3.1 s)] and 1 s for the PBP [*n*=88, 90% CI (0.3 s, 2.3 s)]-treated samples. The data were aggregated from three different embryos for each treatment. *P<0.05; ***P<0.001. The horizontal line represents the median, the boxes represent the 25th and 75th percentiles, the vertical bars represent the 9th and 91st percentiles, and the crosses represent outlier data points.

We also tested the deflection amplitude of the junction in the posterior region parallel to the A-P axis. These junctions, which are characterised by less phospho-myosin light-chain accumulation than junctions oriented perpendicular to the A-P axis, also showed larger deflections, reflecting lower tension (Fig. 3A,C). The latter results were obtained from measurements in only two embryos, but are directly in line with an earlier set of experiments, where instead of the pull-and-release protocol, a sinusoidal movement of the optical trap across the junction was used. This comprises a much larger set of measurements (Fig. S5). The latter experiment showed a significant larger difference in deformation of junctions in the posterior epiblast oriented parallel to the A-P axis than junctions oriented perpendicular to the A-P axis in stage EGK XII-XIV embryos.

Together, these results show that junctions that are known to contain higher levels of active myosin, i.e. junctions in the posterior of the embryo aligned perpendicularly to the A-P axis, also show higher tensions then junctions of cells parallel to the A-P axis.

Further results obtained with the sinusoidal perturbation protocol showed that junctions of cells in the posterior embryonic region in very young embryos (stage EGXI-EGXII), before the onset of motion of cells in the epiblast and the formation of the active myosin cables, were more easily deformed than the junctions that were in the process of starting to form a streak (Fig. S6). These measurements provide further support for the hypothesis that differences in tension drive differences in cell behaviours, such as directional cell intercalation.

### Effect of myosin inhibitors

Our previous work has shown that myosin II plays a key role in the execution of direction cell intercalation and streak formation (Rozbicki et al., 2015). Inhibitors of myosin II, as well as siRNA-mediated downregulation of myosin IIa and myosin IIb, resulted in inhibition of directional cell intercalation and streak formation. We also found that inhibition of myosin I family members through inhibitors or specific siRNAs also resulted in a strong reduction of myosin II activity, as measured by inhibition of the formation of cables of myosin light chain phosphorylation, which resulted in a complete inhibition of directional cell intercalation and a block of streak formation. To investigate the roles of myosin I and myosin II in the generation of junctional tension in the mesendoderm cells, we inhibited the activity of these myosins using the class-specific inhibitors pentachloropseudilin (PCP), which specifically inhibits members of the myosin I family, and pentabromopseudilin (PBP), which is a strong inhibitor of myosin II (Chinthalapudi et al., 2011; Fedorov et al., 2009; Rozbicki et al., 2015). We used a concentration of 10 µM for both inhibitors (Fig. 4).

We measured the response of cell-cell junctions perpendicular to the A-P axis in the posterior area of embryos in embryos that were treated with these inhibitors compared with control-treated embryos (Fig. 4A). Lifting the embryos followed by the application of a small amount of liquid under the embryos did not affect the junctional tension, as the deformations measured were not statistically significantly different from measurements of deformations in control embryos that were not manipulated (compare Fig. 3B, left panel, and Fig. 4A, *P*=0.41). However, we did measure a statistically significant larger junctional deformation after the embryos were treated with either a myosin I (PCP) or the myosin II (PBP) inhibitor, compared with control-treated embryos. After treatment with PBP, the maximum deformation increased from 0.41 µm in the control data to 0.71 µm after inhibitor treatment. The treatment with PCP also caused the maximum deformation to increase to reach 0.62 µm, results that were statistically highly significant. These experiments show that myosin II activity is a major determinant of junctional tension. They furthermore show that inhibition of myosin I activity results in a large decrease in junctional tension, consistent with its reported effect on myosin II cable formation.

When we extracted the relaxation time from the fitting of the junctions, we observed small but highly significant difference between the distributions, with a median of 1 s for the PCP- and PBP-treated cases compared with the 0.7 s measured in the case of control junctions (Fig. 4B). The relaxation times in the control-treated embryos showed no significant differences from those of non-treated control embryos (compare Fig. 3B and Fig. 4B, *P*=0.9)

The fact that the combined data from all experiments best fitted a Maxwell viscoelastic model (Fig. S8) suggested that the duration of the perturbation will affect the irreversibility of the junction deformation, as was recently very clearly demonstrated during germband extension in *Drosophila* (Clément et al., 2017). This prompted us to see whether this effect could be detected in our measurements performed thus far. Under our experimental conditions, junctions are lost from the trap at various times, depending on trap stiffness and junction tension. We therefore calculated the irreversibility index obtained by dividing the distance of the junction at its time of release from the trap relative to its original position by the position attained at infinite time, as extrapolated from the fit to the viscoelastic model, and analysed this irreversibility index (Fig. S7A) as a function of perturbation time (Fig. S7B). The results show that, for control junctions, only pulling durations of more than 5 s produced a detectable effect. At shorter times, most junctions would revert to their original position. However, after application of the myosin inhibitors PCP and PCB, the irreversible deformations became measurable at shorter pulling times (Fig. S6B,D) and were similar to deformations in junctions in central locations of the epiblast (Fig. S6B,C). As the irreversible deformation at long times is given by 
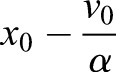
, as can be seen from Eqn 5 in the Materials and Methods, these findings are in line with a reduced tension that is proportional to the initial velocity and an increased time constant, and/or a combination of both in junctions that contain less myosin. In summary, these measurements show that junctional tension in mesendoderm cells is higher than in other epiblast cells of the embryonic area and that tension is higher in mesendoderm cell junctions that are aligned perpendicular to the A-P axis along the direction of contraction. The measured tension patterns mirror the observed distribution of phospho-myosin light chain at these stages of development well, and inhibition of myosin II activity results in reduced tension. Taken together, these results strongly support the notion that the observed phospho-myosin light-chain patterns are good indicators of tension in the early gastrulation stage chick embryo.

## DISCUSSION

While optical tweezers are an established tool for cell and molecular biology, and biophysics, only recently have they have been used in living organism, such as *Drosophila* embryos (Bambardekar et al., 2015) and zebrafish (Favre-Bulle et al., 2017; Johansen et al., 2016). The application of optical tweezers for the manipulation of cell junctions in living organism was first reported by Lenne and co-workers, where it was applied to measure the tension in cell junctions in the gastrulating *Drosophila* embryo (Bambardekar et al., 2015). Our experiments in the chick embryo have shown that these optical tension measurements are possible in more-complex systems. In our studies, it is clear that cellular vesicular organelles in proximity of the junctions become trapped by the tweezers and these are the main objects pushing the junctions. As the size distribution of the vesicles is not uniform, this causes the trap stiffness to vary between measurements, as it depends on the physical properties of the vesicles. For an absolute measurement of the tensions, we would need to calibrate the trap stiffness for each organelle trapped before each measurement. There are techniques that offer active tweezer calibration *in vivo* (Tolić-Nørrelykke et al., 2006), but they rely on forward scattering interferometry, which is extremely challenging in thick scattering media such as the chick embryo sample. Modification of the system to work in back-scattering mode should be possible for future work (Huisstede et al., 2005; Volpe et al., 2007).

Even without quantitative force measurements we have shown that, despite the variance in stiffness introduced by trapping organelles, we can still measure significant differences in tension of populations of junctions in different areas of the chick embryo. Junctions of mesendoderm precursors in the posterior area of the embryo aligned perpendicular to the A-P axis were deformed on average significantly less than junctions aligned in the same direction in the central area of the embryo, showing that the cell junctions in the posterior area are under higher tension. Junctions in the posterior mesendoderm aligned perpendicular to the A-P axis, in the direction of tissue contraction, showed significantly higher average tension than junctions of mesoderm cells aligned in perpendicular direction, along the A-P axis. Furthermore, the observation that application of myosin inhibitors resulted in a significant loss of junctional tension is in line with the hypothesis that the observed phosphorylated myosin II cables are responsible for the differences and alignment of junctional tension in the mesendoderm. Our experiments further confirm the role of myosin I in the generation of tension in cell junctions in the chick embryo, likely through an effect on myosin II accumulation that we have described previously (Rozbicki et al., 2015). These observations support the hypothesis that myosin-generated differential tension drives the observed directional intercalation of mesendoderm cells in the early pre-streak embryos. These findings further stress the great conservation of the cell biological mechanisms underlying and coordinating gastrulation in the chick embryo with the detailed cell behaviours underlying *Drosophila* germband extension, where it has been convincingly shown that junctional myosin accumulation increases junctional tension, underlying directional intercalation during germband extension (Bertet et al., 2004; Clément et al., 2017; Collinet et al., 2015; Fernandez-Gonzalez et al., 2009; Rauzi et al., 2010). In the chick embryo, it remains to be shown how the myosin cables form and which mechanisms are responsible for their global orientation. In *Drosophila*, it has been suggested to be under control of an A-P-dependent chemical patterning system, possibly mediated by a combinatorial code of Toll receptors (Paré et al., 2014). We suggest that the formation of oriented myosin cables may involve a tension-dependent feedback mechanism, where myosin-mediated contraction of a given junction results in increased tension in neighbouring junctions that in turn results in myosin accumulation in those junctions, thus resulting in the long-range orientation of myosin cables then driving directional intercalation. Investigating this will require directional perturbation experiments to be performed on much longer time scales as well as suitable *in vivo* indicators of myosin activity (Harris et al., 2013).

In all experiments, we observed cases where the junctions did not return to their original rest position during the time frame of the measurements. In line with this, we have found that a simple Maxwell viscoelastic model, with only a few parameters, fits our experimental data best. This agrees with measurements in the gastrulating *Drosophila* embryo (Clément et al., 2017). We have used this fitting to obtain the relaxation times with which the junctions relax after release from the trap. We did not observe a significant change in the relaxation time when measuring junctions in different areas of the embryos. There was, however, a modest increase in relaxation time after treatment with the myosin I and myosin II inhibitors. This relaxation time is a combination of a time constant deriving from the viscoelastic properties of the junctions themselves, *T*, and one deriving from the viscosity of the cytosol, τ: 
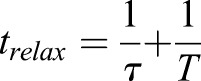
 (see Materials and Methods). We assume that the properties of the cytosol remain invariant between the different measurements and that the observed differences in relaxation times are due to differences in the junction viscoelastic time constant.

The degree of irreversible deformation depends on the dissipation time constant. In *Drosophila* it has been shown that this dissipation constant is around 50 s and that this time constant is highly dependent on actin dynamics (Clément et al., 2017). In our experiments, we deformed the junctions for only a relatively short time, but we observed an irreversible deformation junction at longer deformation times. The effects were more noticeable when myosin activity was inhibited, suggesting that myosin might have role in setting the dissipation time constant. Myosin, besides its motor activity, plays a strong role in crosslinking of actin filaments. Furthermore, changes in contraction of the actin network do affect actin filament turnover (Sonal et al., 2019). These activities could all affect the dissipation time constant. These effects will require further characterisation using a greater range of systematic deformation times, as in the *Drosophila* case. Extending the duration of the experiment would allow measurement of the recoil of the junction experimentally, instead of using the extrapolated values from the Maxwell model fit.

Finally, our findings strongly support the use of optical tweezing methods to measure relative junctional tension in a non-destructive manner and show that these measurements can be used to obtain valuable novel insights into key mechanical characteristics of complex developing systems, such as the early chick embryo. Future investigations will focus on the *in vivo* determination of the trap stiffness, to obtain an absolute value of the junction tensions and on a better characterisation of dissipation characteristics of the junctions in different parts of the embryo, as well as the direct and indirect contribution of various myosin classes on tension generation.

## MATERIALS AND METHODS

### Trapping and imaging

We performed all experiments reported here with a custom-built inverted fluorescence microscope using an infrared trapping laser (Lee et al., 2007). Fig. S1 shows a diagram of the experimental system. The sample was first imaged with a low-magnification microscope in order to locate the area of interest in the embryo (Fig. S1). A custom-made LED ring illuminated the sample with white light at a 45° angle, while images were collected with a 4× microscope objective (Zeiss Achroplan, air immersion, NA=0.1, W.D.=11.1 mm) in reflection mode. For fluorescence excitation of the EGFP in the cell membrane, a 100× microscope objective (Nikon CFI Apochromat TIRF, oil immersion, NA=1.49, W.D.=0.12 mm) working in epifluorescence mode was used in combination with a 488 nm excitation laser (488 Sapphire SP, Coherent). The images were collected using a scientific CCD camera (Hamamatsu Orca flash 4.0). Excitation and emitted fluorescence light were separated by a dichroic mirror (Chroma ZT488rdc) under the microscope objective and an edge filter (Semrock BLP01-488R-23.3-D) before the camera. The same 100× microscope objective was used to generate the trap by focusing an infrared laser (wavelength 1070 nm, ytterbium doped fibre, IPG Photonics). An additional dichroic mirror (Thorlabs DMSP950) and a bandpass filter (Chroma ET750sp-2p8) eliminated the backscattered reflections of the laser onto the camera. The trapping laser was moved using a piezo-actuated mirror (Thorlabs POLARIS-K1S2P) located in a plane conjugate with the back focal plane of the microscope objective. The piezo-actuated mirror and the camera were hardware triggered (National Instruments data acquisition card NI PCI-6251 with connector box SCB-68A) using a Matlab script to ensure that every frame could be assigned a specific trap location. Before every experiment, we removed the bandpass filter and recorded the backscattered reflection of the trap onto the camera to infer the trap position during the experiments. All experiments were performed at 750 mW laser power, as measured in the image plane. The trap moved of a total distance of 2.6 µm over a time interval of 5 s and was kept in the final position, while still on, for 2.5 s and then turned off for another 2.5 s. During the experiment, the embryos were kept at 37°C in a custom-built incubator chamber.

### Embryo sample preparation

A membrane-localized GFP transgenic chicken line was used in all experiments (Rozbicki et al., 2015). Fertilised eggs were obtained from the national Avian Research Facility at the Roslin institute in Edinburgh, UK (www.narf.ac.uk/chickens/transgenic.html).

The embryos were isolated and allowed to develop in EC culture at 37°C (Chapman et al., 2001). For most experiments, we used embryos with a well-defined Koller's Sickle, where the hypoblast had closed (EGK XIII-XIV). For some experiments, we used embryos at earlier stages of development, before closure of the hypoblast EGK (XI-XII) (Eyal-Giladi and Kochav, 1976). For all experiments, the embryos were mounted with the epiblast layer facing downwards on a Willco 3 cm glass bottom cell culture dish and covered with 2 ml of low viscosity light silicon oil (viscosity 5cSt, Sigma, 317667) to prevent drying out of the embryo. The experiments were performed within 2 h of mounting the embryos.

Sets of position-dependent measurements in the posterior and middle positions were performed on the same embryo. Typically, 10 junctions oriented perpendicular to the A-P axis were measured in both positions within an experimental time of 1 h.

### Myosin inhibitors experiments

Stock solutions (10 mM) of the myosin inhibitors pentabromopseudilin (PBP) and pentachloropseudilin (PCP) in DMSO were diluted to 10 µM in 0.9% NaCl for use in the experiments. The myosin inhibitor experiments consisted of sets of measurements before and after addition of the myosin inhibitors. Embryos (stage EGK XIII-XIV) were mounted in a glass-bottomed petri dishes and trapping experiments were performed for 30 min on junctions located in the posterior area of the embryo aligned perpendicularly to the A-P axis. The embryos were then carefully lifted from the glass bottom and treated with 25 μl of a 10 µM PBP/PCP solution. After an incubation time of 20 min, the trapping experiments were repeated in the posterior area of the sample. Control experiments consisted of sets of measurements before and after addition of a 0.1% DMSO solution. Analysis of the data showed that lifting of the embryo had no significant effect on the tension measured. Analysis of the data showed no significant correlation between the deformations measured and the duration of the experiments lasting up to 2 h in control experiments.

### Data processing and data analysis

A custom MATLAB GUI was implemented to control the components of the set-up and to trigger the camera. The images collected through MATLAB were saved as archival format video (mj2), while a .mat file stored the instrument settings of the measurements. For the analysis, we applied a Gaussian smoothing filter and stretched the contrast for each frame. Through a custom MATLAB function, we produced kymographs for every location along the junction, generating a stack of kymographs. For each kymograph, we identified the location of the junction: we observed that performing a Gaussian fit of the fluorescence intensity along the kymograph lines to achieve subpixel resolution was not effective for our data and failed to identify the junction position because of the low signal-to-noise level and the presence of scattered light from the organelles in proximity of the junction. Therefore, we adopted a modified version of a seam-carving algorithm. Seam-carving algorithms are designed for content-aware resizing by removing the paths in an image that have minimal variation (Avidan and Shamir, 2007). In a greyscale image, these paths correspond to the shortest paths between the first row and last row of the image, weighting pixel values on the greyscale. Our modified seam-carving algorithm finds the shortest path for each kymograph in the stack, weighting both on the greyscale value of each pixel in the current kymograph and on the neighbour pixel of the previous and subsequent kymograph in the stack. This approach enhances the precision in determining the junction location at each frame for our datasets. Fig. S3 shows a comparison between using Gaussian fittings and adopting the seam-carving algorithm to determine the junction locations. Finally, we identified the kymograph associated with the highest deformation of the junction, we converted the results in µm and s, and we extracted the value of the maximum deformation. By plotting the deformation of the junction against time, we concluded our analysis by fitting the data with the *fit* function in Matlab (with the Robust mode on) using as ‘fittype’ the equation derived from the Maxwell viscoelastic model. The data were aggregated and compared.

Initial analysis of the distribution of aggregated data from comparable samples using the Anderson-Darling test (adtest function in Matlab) showed that the data failed the test of normal distribution. Therefore, we used the non-parametric Wilcoxon rank sum test (ranksum function in Matlab) to test for significance of differences between samples.

### Viscoelastic model

To describe the dynamics of cell-cell junctions, we used a Maxwell viscoelastic model. This model describes the junction as if it were a purely elastic element of the elastic constant *E* and a purely damping element of the damping coefficient *η* (Fig. S7).

When the junction is deformed by a strain *x*, it is subjected to a tension force *F*_*j*_ opposing the deformation:(1)
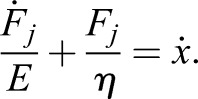


During the optical manipulation, the balance of forces reads as:(2)
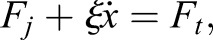
where*ξẋ* is the drag in the cytosol for an object (the manipulated junction) moving with speed *ẋ* and *F_t_* is the optical force.

As we could not characterise the stiffness of the optical tweezers, we used the model to fit the relaxation time after switching off the trapping laser. When the tweezers are off, the tension is balanced only by the drag in the cytosol opposing the return of the junction to the rest position:(3)
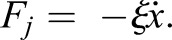


Using this definition of the force with Eqn 1, we can derive the differential equation:(4)
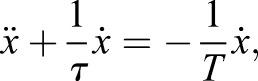
where *τ*\tau = *η* /*E* represents the relaxation time of the junction and *T*=*ξ* /*E* is the relaxation time of the cytosol.

The solution that describes how the junction moves back to its rest position is a single exponential curve:(5)
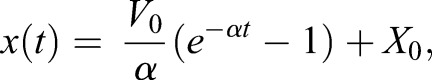
where 
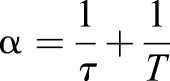
, *X*_0_ the position of the junction at *t*=0 (release from the trap) and V_0_ is its initial velocity, immediately after release from the trap.

We used the ‘fit’ function in Matlab with ‘Robust mode’ on to fit the data with a solid linear solid model, and with a Kelvin-Voigt model, in addition to the described Maxwell model; however, we found that the Maxwell model outperformed the other fits for accuracy (Fig. S7).

## Supplementary Material

Supplementary information
