# Supplementary material for: Measurement of junctional tension in epithelial cells at the onset of primitive streak formation in the chick embryo via non-destructive optical manipulation
Source: Development. 2020 Feb 4;147(3):dev175109. doi: 10.1242/dev.175109 (PMC7033729; doi:10.1242/dev.175109)
Supplement: Supplementary information [file develop-147-175109-s1.pdf]

SUPPLEMENTARY INFORMATION

A

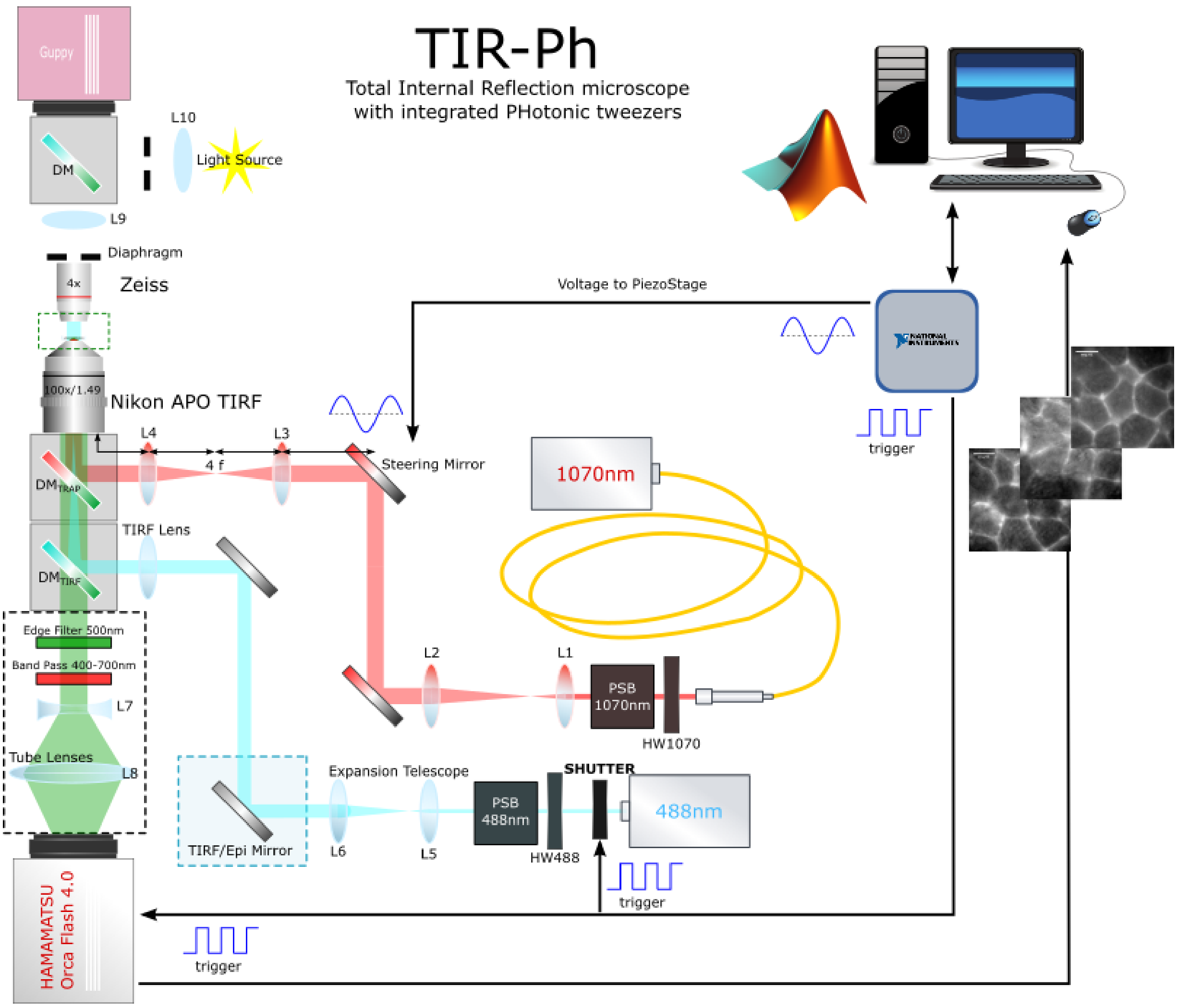

B

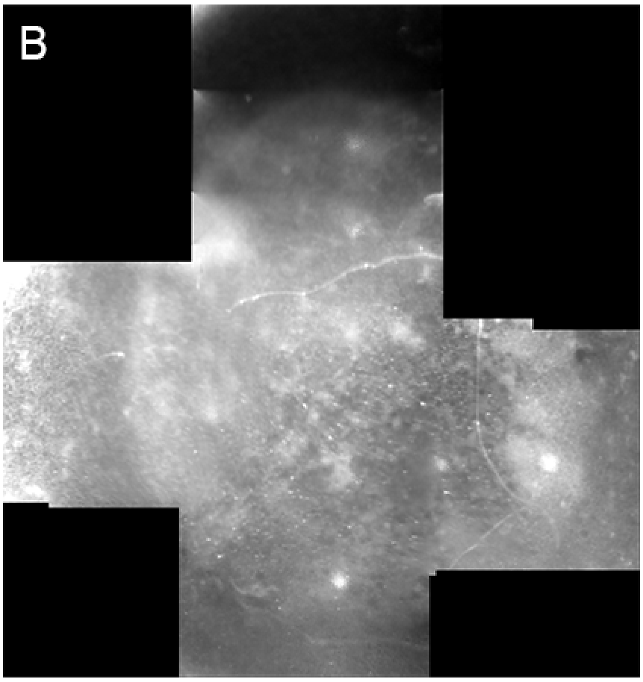

Figure S1. (A) Optical setup combining epifluorescence microscopy and optical tweezers. For details of setup see materials and methods

(B) Example of low magnification image of the chick embryo taken with the Guppy camera. Low magnification images were used to orientate in the embryo.

Development • Supplementary information

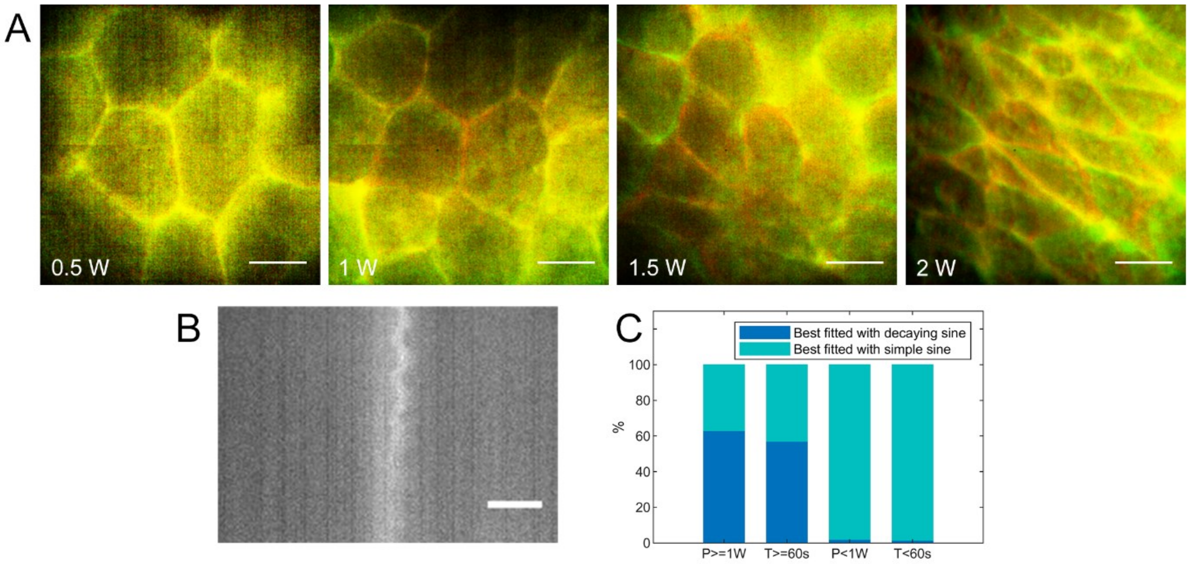

Figure S2. Power damage on chick embryos junctions.

A: False colour images of a chick embryo subjected to different laser powers: in red the embryo at time t=0, before the laser is turned on; in green the same embryo observed after the laser was left on for 30 seconds. For laser powers <1 W, there is no visible damage: the first frame and the last frame overlap. For power = 1 W, there is minimal damage. At powers >1.5W, major damage is observed: the junctions in the embryo relax and a long range deformation is observed.

B: shows the kymograph of a junction being subjected to sinusoidal motion of the trapping laser (see Fig. S5). The laser power was 1W and the duration of the measurement was 60 seconds. The kymograph shows that the junction follows the trap movement initially, but the amplitude of deformation reduces over time.

C: This behaviour can be fitted by using an exponentially decaying sine function. We compared junctions subjected to sinusoidal motion with different parameters (power <1 W, power >= 1W, duration <60s, duration >=60s) and verified whether they would be best-fitted by a decaying sine wave (C). We observed that the decay behaviour was predominantly observed after longer exposure to the laser and at higher powers. This suggests that the junctions change during long exposure and or higher power.

Based on these findings we chose a constant power of 750mW. At this power the embryos showed no visible damage, while we were able to move a higher percentage of junctions than by using lower power (i.e. 500mW).

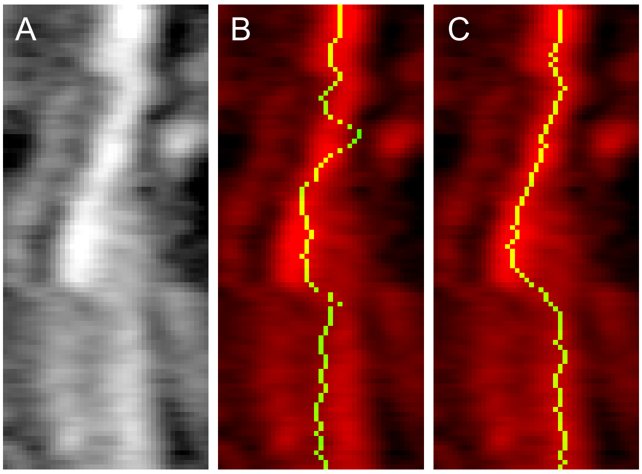

Figure S3. Gaussian fit vs seam carving algorithm comparison. **A:** shows a junction kymograph. **B-C:** False colour images with the derived junction positions overlaid in yellow over original kymograph: in (B) the junction positions were extracted by fitting each line profile of the kymograph with a Gaussian curve; in (C) the junction positions were extracted by using an implementation of the seam carving algorithm. The sequence (A-C) shows that the seam carving algorithm better reproduces the position of the junction than that obtained by using a Gaussian fit.

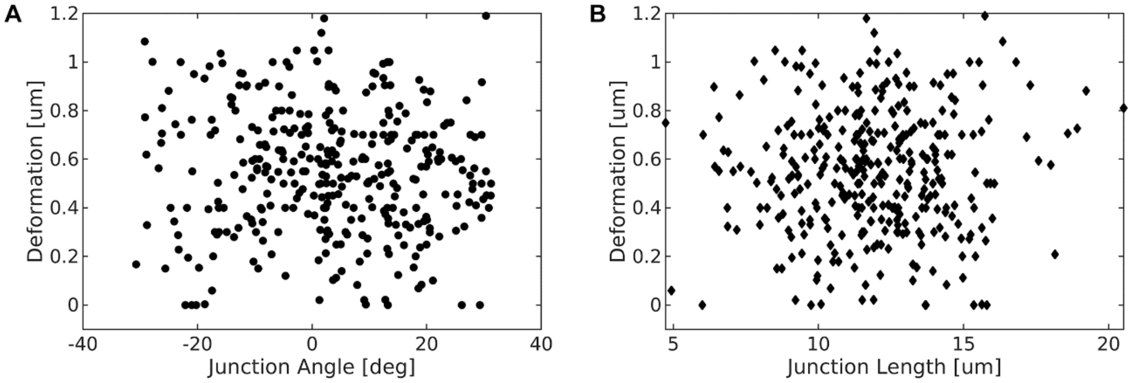

Figure S4. A: Absence of a significant correlation between deflection angle and junction deformation. Correlation Coefficient r =-0.07, p=0.19.

B: Absence of a significant correlation between junction length and junction deformation. Correlation Coefficient r =0.04, p=0.45.

Development • Supplementary information

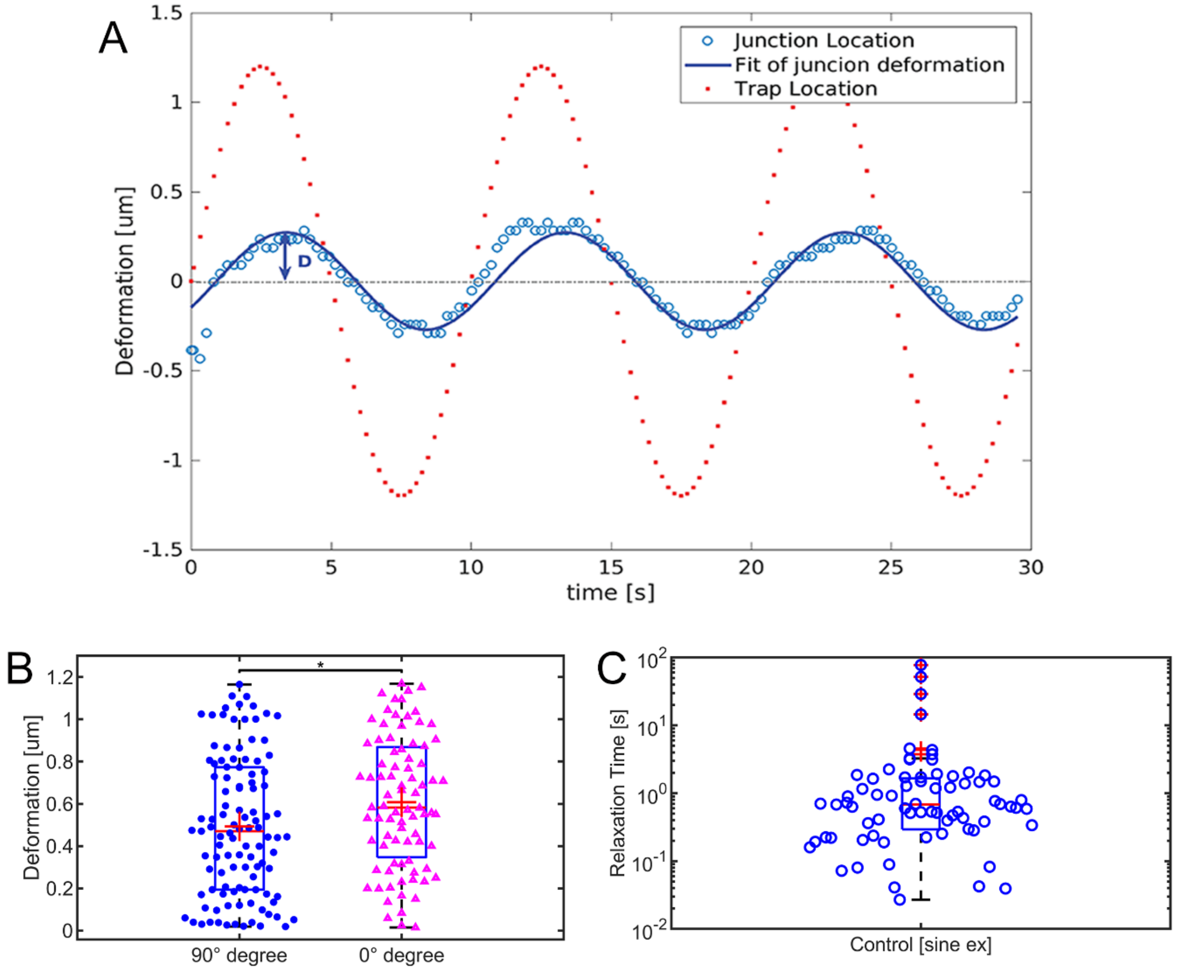

Figure S5 Deformation of junctions perpendicular and parallel to the A/P axis.

A: Example of position of the junction over time (blue empty circles) with its fit (blue line) compared with the position of the trapping laser (red dotted line) for sine wave pulling experiments. In these experiments the trapping laser moved back and forth with a sinusoidal motion rather than the pull and release approach used in the majority of the experiments described in the main text. The trap oscillated with a sinusoidal motion amplitude of 2.6 μm and a frequency of 0.1 Hz for a duration of 30s. We extracted the movement of the junction over time by applying the seam carving algorithm to the kymograph of the junctions. The position of the junction over time was fitted with sine function and we extracted amplitude, phase and period.

B: Boxplot and distribution of the maximum deformation of junctions as fitted from the sine wave experiments in cell junctions with different alignment respect to the A-P axis: junction perpendicular to the A/P axis, and therefore aligned to the super-cellular

Development • Supplementary information

Myosin cables (blue filled circle, n=109 collected over 4 embryos) had a median deformation of 0.45 µm, while junctions parallel to the A/P axis (pink empty triangles, n=84 collected over 4 embryos) had a median deformation of 0.6µm. * indicates p-values <0.05

C: From the phase shift between the trap and the junction movement, it is possible to estimate the relaxation time of the system. According to viscoelastic models, $t_{relax} = \tan(\varphi)$, where φ is the phase shift expressed in degrees. We measured a relaxation time of 0.68s for junctions perpendicular to the A/P axis, which is in good agreement with the values measured by using the viscoelastic fitting in the pull & release experiments.

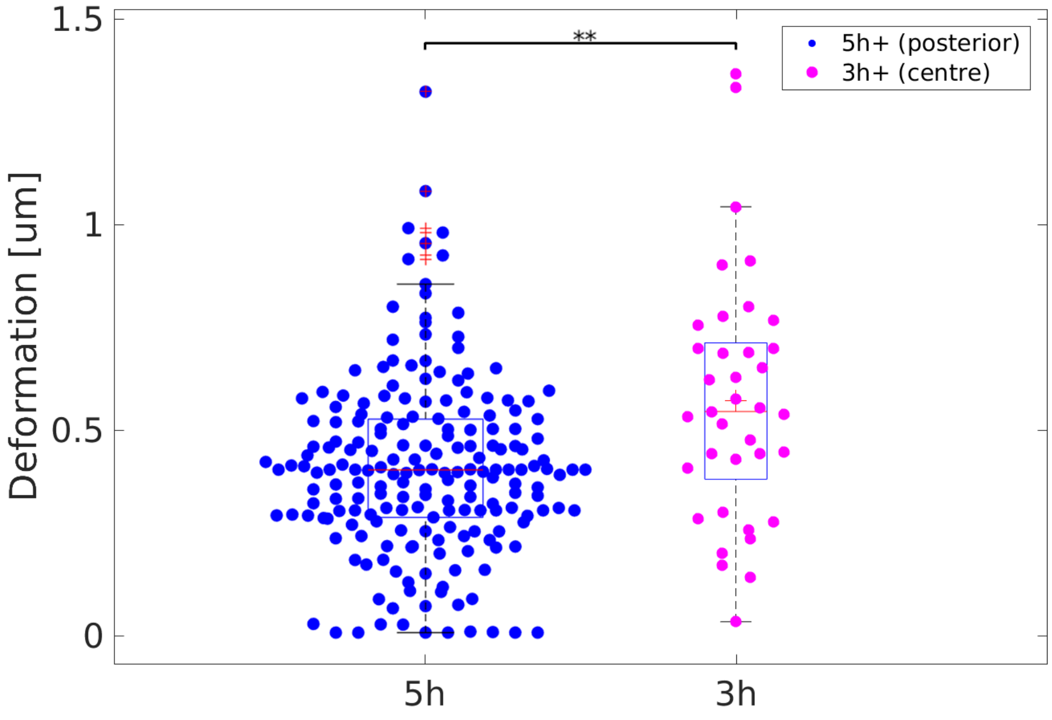

Figure S6. Deformation of junctions in chick embryos of different age.

Boxplot and distribution of the maximum deformation of junctions measured in the posterior of 5 h embryos (EGK XIII-XIV) embryos (blue filled circle, n=203) and in the

Development • Supplementary information

centre of 3h (EGK XI-XII) embryos (pink circles, n=37). Median values for the deformation are 0.39µm 0.59µm for the 5h old and the 3h old embryos respectively.

The data for the 5h old embryos were aggregated from ten different embryos (Control embryos in the main text), while the data for the 3h old embryos were aggregated from two different embryos each. ** indicates p-values <0.01.

The significant difference between the two dataset reinforces the hypothesis that junctional tension increases with the presence of super-cellular Myosin II chains.

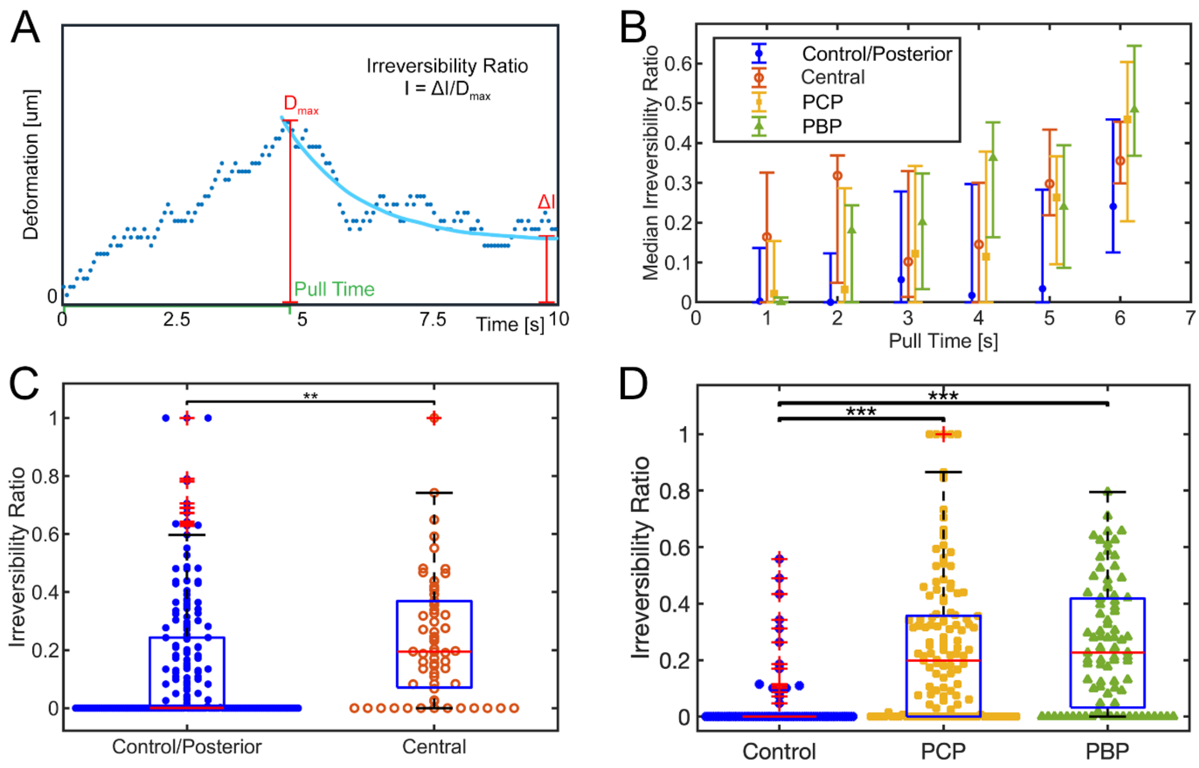

Figure S7. Irreversibility Ratio

A: Cartoon of the definition of irreversibility ratio: the ratio between the position of the junction after being pulled at infinite time ΔI (as measured through the Maxwell model) and the maximum deformation of the junction D_{max} (red lines),

B: Median Irreversibility ratio as function of pulling time. The data are aggregated in intervals of pulling time of 1 second. Pull times are defined as the time which the

Development • Supplementary information

junction is released from the tweezers (Green line in A). Error bars represent the 25 and 75 percentiles.

C-D: Boxplot and distribution of the irreversibility ratio of controls (blue filled circles, n=203) compared with that of junctions measured in the central area of the embryo (C, empty circles, n=57) and compared with that measured in embryos treated with Myosin inhibitors PCP (D, yellow squares, n=132) or PBP (green triangles, n=88. ** indicates p-values <0.01; *** indicates p-values < 0.001.

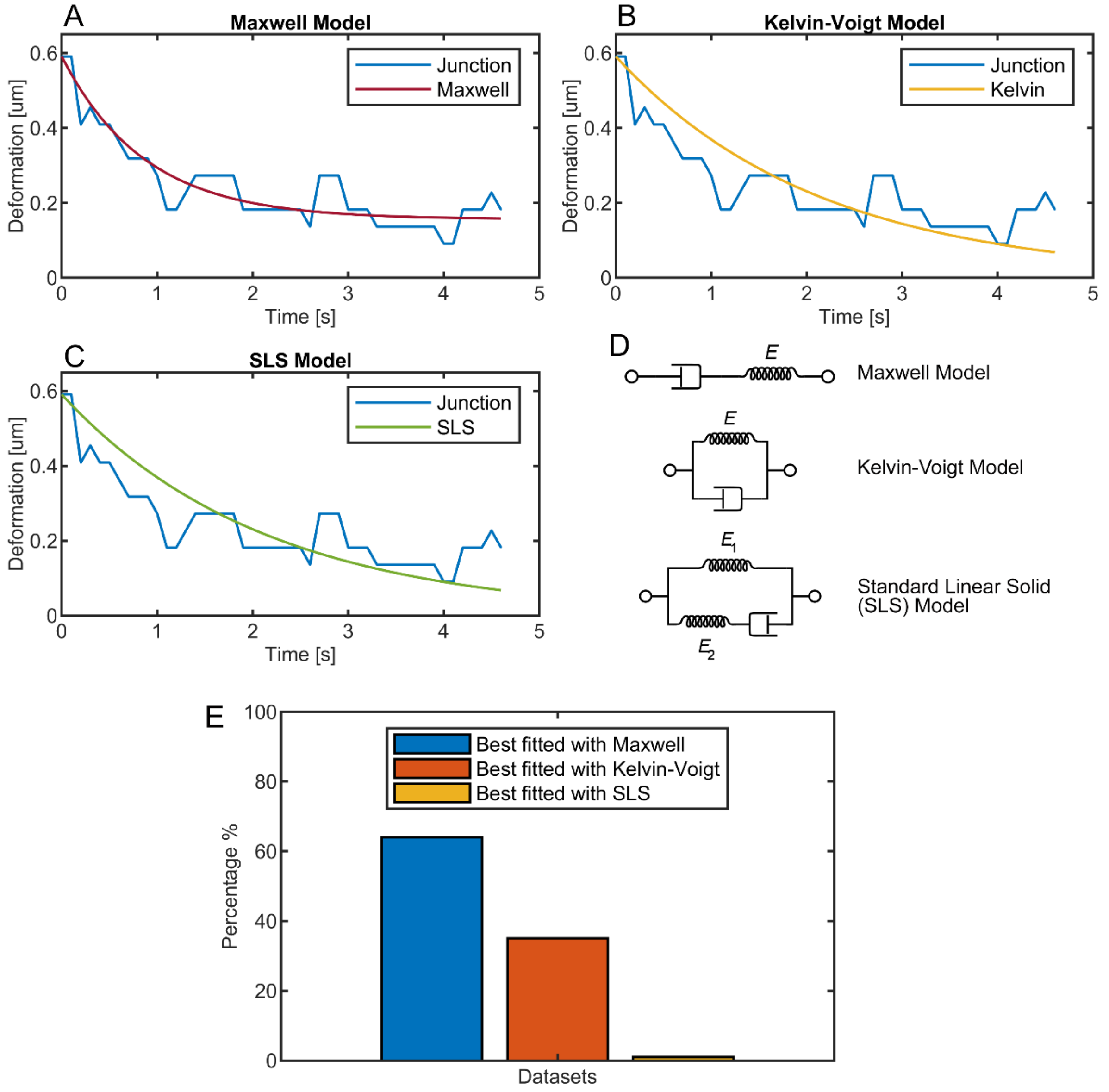

Figure S8. Comparison of goodness of fit of three visco-elastic models

Development • Supplementary information

A-C: Example of a junctional relaxation kinetics (blue line) fitted with Maxwell model (A), with Kelvin-Voigt model (B) and with SLS model (C). To decide the best fit, we compared two parameters, the adjusted R-squared and the absolute error on the measured variables. For the junction in the example, the Maxwell fitting presented an Adjusted R-squared of 0.84 and absolute error on fitting variable of 0.07; the Kelvin-Voigt fitting presented an R-squared of 0.38 and absolute error on fitting variable of 0.57; the SLS fitting presented an R-squared of 0.35 and absolute error on fitting variable of 1E16. We concluded that the best fit was obtained by using the Maxwell model fitting.

D: Schematic representation of the three models compared.

E: By fitting our datasets for the push & release experiments with all three models, we observed that the Maxwell model best fits most of our data. The major difference between the Maxwell and the Kelvin-Voigt model is that the first accommodates the irreversibility of the junction deformation, while the second provides a fitting that will always return to the rest position at 0. The data are the based on analysis of 203 junctions in control experiments of various embryos.

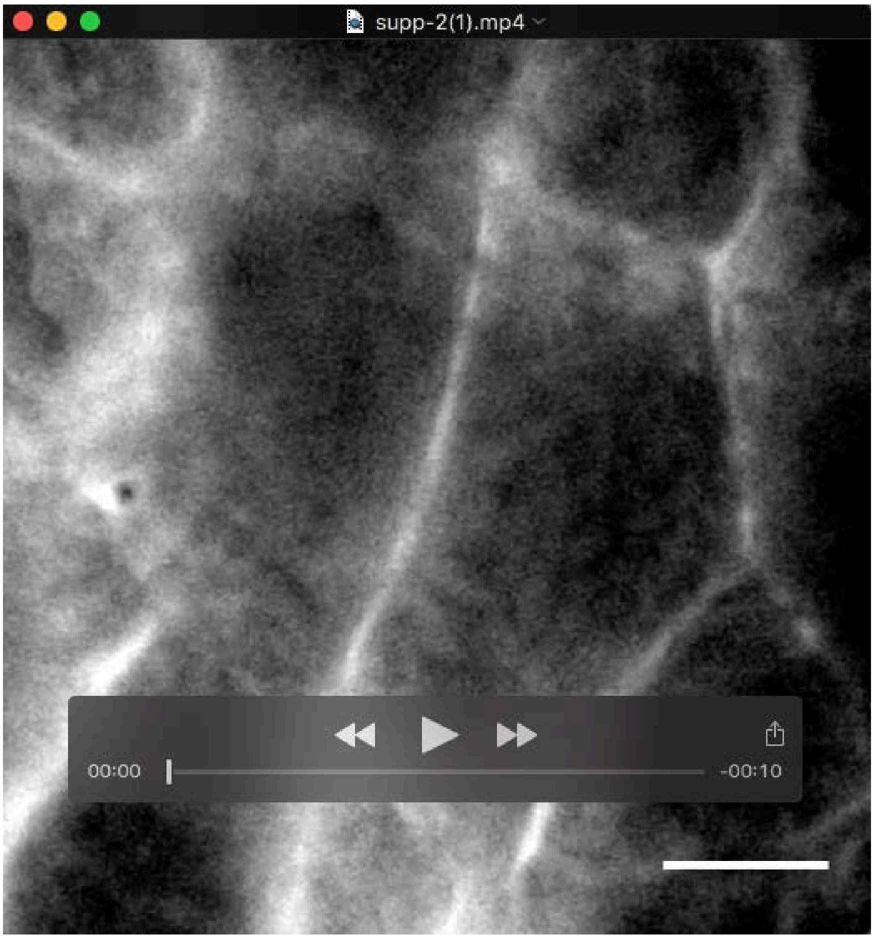

Movie 1: Example of Pull & Release experiment showing the deformation of a junction. The video was acquired at a frame rate of 16.4 frames / second, and it is reproduced at 16 frames / second. Some material inside the cells can be seen to follow the movement of the optical tweezer. We believe that is material consist of a vesicular organelle (possibly several smaller organelles) trapped by the laser and that this is pushed against the junction leading to its deformation. Scale bar is 5 um.

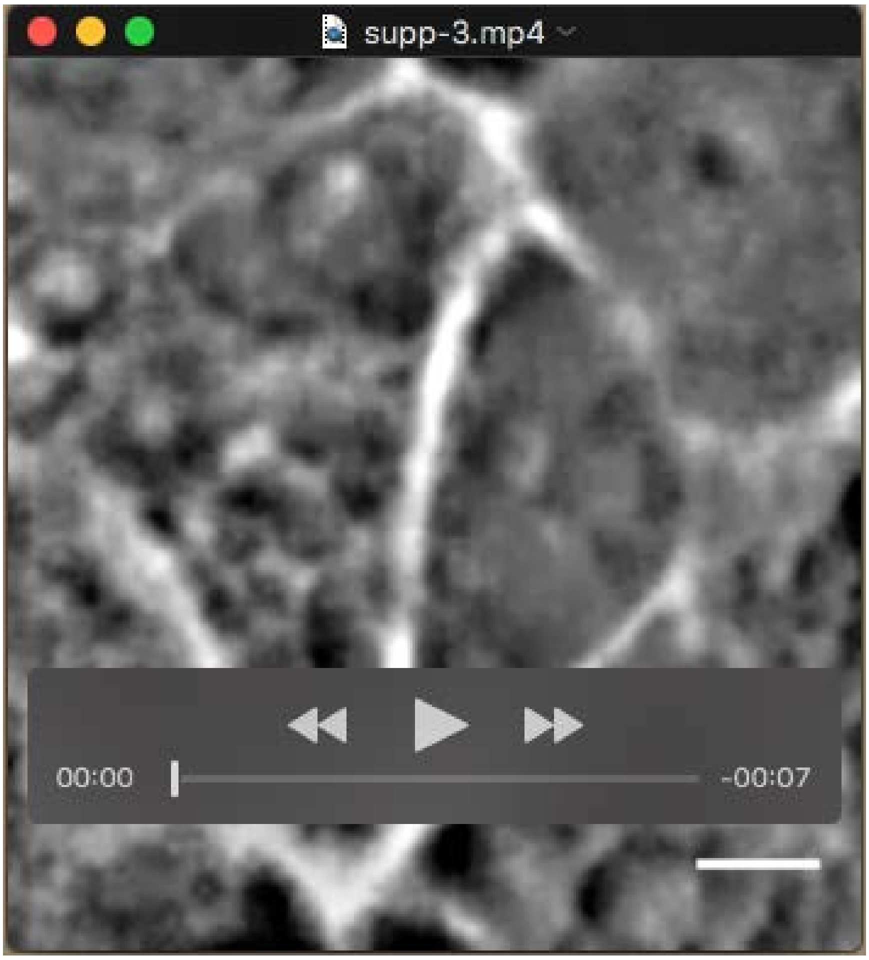

Movie 2: Example of Pull & Release experiment showing the deformation of a junction when pushed by an organelle. The video was acquired at a frame rate of 10 frames / second. The video rate is 14 frames / second. Some material inside the cells that appear to flow that may represent other smaller organelles can be seen to follow the movement of the optical tweezer. Scale bar is 3 um.
